# Role of Forkhead Box Protein O1 (FoxO1) in Stroke: A Literature Review

**DOI:** 10.14336/AD.2021.0826

**Published:** 2022-04-01

**Authors:** Sichao Guo, Ruchi Mangal, Chaitu Dandu, Xiaokun Geng, Yuchuan Ding

**Affiliations:** ^1^Luhe Institute of Neuroscience, Beijing Luhe Hospital, Capital Medical University, Beijing, China.; ^2^Department of Neurology, Beijing Luhe Hospital, Capital Medical University, Beijing, China.; ^3^Department of Neurosurgery, Wayne State University School of Medicine, Detroit, MI 48201, USA

**Keywords:** transcription factors, BBB, apoptosis, oxidative stress, gluconeogenesis, inflammation

## Abstract

Stroke is one of the most prevalent causes of death around the world. When a stroke occurs, many cellular signaling cascades and regulators are activated, which results in severe cellular dysfunction and debilitating long-term disability. One crucial regulator of cell fate and function is mammalian Forkhead box protein O1 (FoxO1). Many studies have found FoxO1 to be implicated in many cellular processes, including regulating gluconeogenesis and glycogenolysis. During a stroke, modifications of FoxO1 have been linked to a variety of functions, such as inducing cell death and inflammation, inhibiting oxidative injury, affecting the blood brain barrier (BBB), and regulating hepatic gluconeogenesis. For these functions of FoxO1, different measures and treatments were applied to FoxO1 after ischemia. However, the subtle mechanisms of post-transcriptional modification and the role of FoxO1 are still elusive and even contradictory in the development of stroke. The determination of these mechanisms will lead to further enlightenment for FoxO1 signal transduction and the identification of targeted drugs. The regulation and function of FoxO1 may provide an important way for the prevention and treatment of diseases. Overall, the functions of FoxO1 are multifactorial, and this paper will summarize all of the significant pathways in which FoxO1 plays an important role during stroke damage and recovery.

Stroke causes irreparable cell damage and results in severe morbidity and mortality globally [1, 2]. FoxO1 plays a major role in brain damage following a stroke. The structure of the protein may have a large part in this process. The fork head domain was first identified as a 100 amino acid residues of sequence similarity between the Drosophila fork head and the rat HNF-3a proteins, and then it was identified in numerous gene families ranging from yeast to humans [3]. The “O” subclass of the Forkhead transcription factors (FOX) family-Forkhead box protein O (FoxO) functions as a transcription factor that has arisen as a vital regulator of cell fate and function in mammals. The FoxO family contains the evolutionarily conserved fork head box domain, which constitutes a functional unit that is necessary and sufficient for DNA binding. The FoxO family includes four members expressed in nearly all tissues including FoxO1 (FKHR), FoxO3 (FKHRL1), FoxO4 (AFX), and FoxO6 [4-6].

FoxOs are important in many processes, such as reactive oxygen species (ROS) suppression, induction of apoptosis, promotion of survival and longevity, engagement of autophagy and regulation of metabolism. The function of FoxOs is typically governed through post-translational modifications of the proteins, which, in turn, regulate FoxOs subcellular localization and/or transcription [7-9]. Different subtypes of FoxO proteins have different functions in different diseases. FoxO1 is considered to be a representative member of the FoxO family, and has key transcription regulatory activities [6]. FoxO1 is the most widely studied subtype and is known to regulate a wide range of molecular signals in many tissue types, such as liver and brain [10]. FoxO1 has been shown to have numerous effects on a broad range of diseases, including cardiovascular disease, diabetes, and cancer [11]. During cardiac ischemia, FoxO1 is not only present, but also persists throughout the process of cardiac ischemia [12]. It is also an important regulator of aging and longevity [13]. Currently, the role FoxO1 plays in stroke damage will be assessed. Strokes are caused by the occlusion of major cerebral arteries, leading to widespread tissue damage, which may be associated with the FoxO1 activity. This review will discuss all of the significant pathways in which FoxO1 plays an important role during stroke damage and recovery.

## The regulation of FoxO1 post-translational modifications

Post-translational modification is an important way to regulate protein function and control the physiological processes. A series of modifications are involved in the regulation of FoxO1 activity, including phosphorylation [14], acetylation [15], methylation [16, 17], ubiquitination [18, 19], and glcNAcylation [20]. These modifications could affect the subcellular distribution and DNA binding affinity of FoxO1, which in turn modulates its activity [21]. The major post-translational modifications that are studied on FoxO1 after stroke are acetylation and phosphorylation.

In general, phosphorylation promotes nuclear exportation, polyubiquitination, and proteasomal degradation, which presents as an inhibitory effect on FoxO1 activity [21]. FoxO1 can be phosphorylated by phosphatidylinositol 3-kinase/Protein Kinase B (PI3K/PKB or Akt) [22], AMP-Activated Protein Kinase (AMPK) [23], mammalian sterile 20-like kinase 1 (MST1) [24], and cyclin-dependent kinase 1 (CDK1)[25]. Of note, Akt is the primary upstream kinase in FoxO1 signaling transduction pathway regulation and is a negative regulator of FoxO1 [26, 27], including after a stroke. In normal cerebral homeostasis, the serine/threonine protein kinase Akt down regulates the activities of FoxO1 by phosphorylation at three phosphorylation sites- Thr24, Ser256, and Ser319 [28]. After its nuclear exportation, FoxO1 is retained in the cytoplasm, which promotes its proteasomal degradation [29, 30]. In cerebral ischemic injury, however, activation of Akt is suppressed. FoxO1, with its decreased phosphorylation, has increased activity and is able to undergo nuclear translocation and regulate its target genes [31]. It has been reported that protein phosphatase 2A (PP2A) could dephosphorylate FoxO1 in some conditions [32]. Whether PP2A or other pathways could dephosphorylate FoxO1 after stroke needs to be further studied.

Besides phosphorylation, FoxO1 undergoes additional post-translational modifications after stroke such as acetylation. Acetylation of FoxO1 impacts its DNA binding and transcriptional activity [33, 34]. Conversely, acetylated FoxO1 can be deacetylated by sirtuin 1 (SIRT1) [35, 36]. SIRT1 has been shown to play a significant role in neuroprotection against cerebral ischemia by deacetylating and activating FoxO1 [37, 38].

## Pathological Roles of FoxO1 after Stroke

### FoxO1 facilitates cell apoptosis

**Figure 1.**
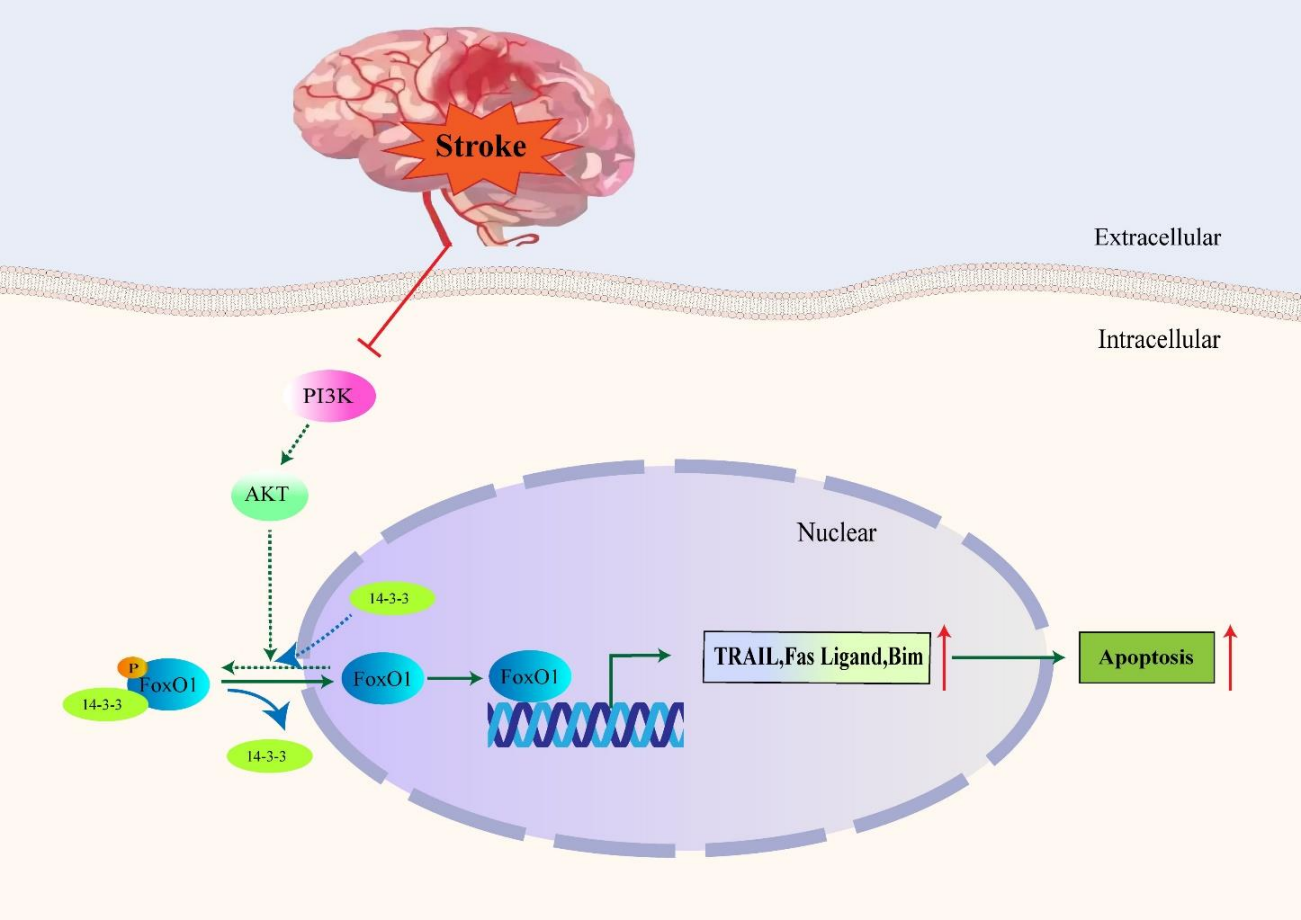
Schematic model for role of FoxO1 in apoptosis. After stroke, activation of PI3K/Akt pathway is suppressed. Then the phosphorylation of FoxO1 and the interaction between p-FoxO1 and 14-3-3 are inhibited which further lead to the nuclear translocation and activation of FoxO1. After FoxO1 is activated, downstream apoptosis pathway including TRAIL, Fas Ligand and Bim are also regulated accordingly. The solid lines represent the pathway that occurs after stroke, and the dotted lines represent the pathway suppressed after stroke.

FoxO1, part of the forkhead family of transcriptional regulators, exerts pro-apoptotic effects on many cell types [6, 39]. Won et al. reported that the mechanisms of cell death were associated with the activation of FoxO1 during cerebral ischemia, which was also the first study of the FoxO family in stroke [40-42]. Activation and nuclear translocation of FoxO1 causes itself and its target genes to translocate from the cytoplasm to nucleus[43]. FoxO1 directly regulates the extrinsic apoptotic pathway through stimulating expression of apoptotic factors. These factors include the Fas ligand, tumor necrosis factor related apoptosis-inducing ligand (TRAIL), and Bim (Fig. 1) [44-46], via a consensus FoxO1 binding site (GTAAACAA) in DNA-binding-dependent mechanisms [28]. Furthermore, downregulation of the brain-derived neurotrophic factor (BDNF) also inhibits PI3K/Akt pathway, promoting further nuclear translocation of FoxO1 during brain ischemic reperfusion injury to induce apoptosis [28]. Overexpression of phosphatase and tensin homolog deleted on chromosome ten (PTEN), results in inhibition of the PI3K/Akt axis and activation of apoptosis pathway [47, 48].

In a study with rats that underwent middle cerebral artery occlusion (MCAO), the knockdown of lncRNA SNHG15 inhibited neuronal apoptosis and decreased the infarct area by targeting the miR-183-5p/FoxO1 signaling activation axis [49]. The knockdown of lncRNA SNHG15 resulted in the downregulation of the expression of FoxO1 and the inhibition of neuronal damage, suggesting a key role of FoxO1 in inducing cell apoptosis. Downregulation and inhibition of FoxO1 leads to decreased neuronal apoptosis, suggesting that it may be an important target of therapeutic intervention.

Further studies proved that the phosphorylation of FoxO1 (p-FoxO1) was retained in the cytoplasm through interaction with another anti-apoptotic factor, 14-3-3. During cerebral injury, both levels of p-FoxO1, along with interactions of p-FoxO1 and 14-3-3, decreased significantly in the injured regions [50, 51]. By blocking the initiation of Fas ligand gene activation and caspase cascade activation, the interaction of p-FoxO1 with 14-3-3 inhibited apoptotic cell death. However, when the binding between p-FoxO1 and the 14-3-3 protein was decreased, FoxO1 was able to undergo nuclear translocation (Fig. 1) [50]. Furthermore, the study showed that the proline-rich Akt substrate of 40-kDa (PRAS40) protein was also identified as a substrate of 14-3-3 binding protein. Overexpression of PRAS40 can promote phosphorylation of Akt/FoxO1 with inhibition of FoxO1 activity, and it can be associated with reduced infarction size in rats that undergo stroke [51].

Another role that FoxO1 plays during cerebral ischemia is repressing the expression of survivin. Survivin is a member of the inhibitors of apoptosis (IAP) gene family, which inhibits the activation of caspase death cascade by acting upstream from the cascade thus halting apoptotic cell death. FoxO1, acting as a repressor protein, represses survivin gene transcription by directly binding to the survivin promoter. During ischemic stroke, the reduced levels of p-FoxO1 may allow for increased FoxO1 translocation from the cytoplasm into the nucleus, thereby increasing the repression on the survivin promoter region and further contributing to cell apoptosis [52, 53].

Prolonged or severe Endoplasmic Reticulum (ER) stress could also induce apoptotic cell death via FoxO1, through activation of multiple ER-specific pro-apoptotic factors including C/EBP-homologous protein (CHOP) [54]. Expression levels of CHOP are very low under physiological conditions, but they are strongly upregulated in response to ER stress caused by ischemic stroke [55, 56]. ER stress mediates dephosphorylation and activation of FoxO1. The activated FoxO1 could then translocate to nuclei of neuronal cells and activate CHOP in a rat model of focal ischemia suggesting a key role of FoxO1 in facilitating ER stress [43]. There are many ways that FoxO1 induces apoptosis, which ultimately contributes to the pathogenesis of strokes. Targeting these pathways may prove to be a useful therapeutic intervention.

### FoxO1 accelerates hepatic gluconeogenesis as the consequences of hyperglycemia

**Figure 2.**
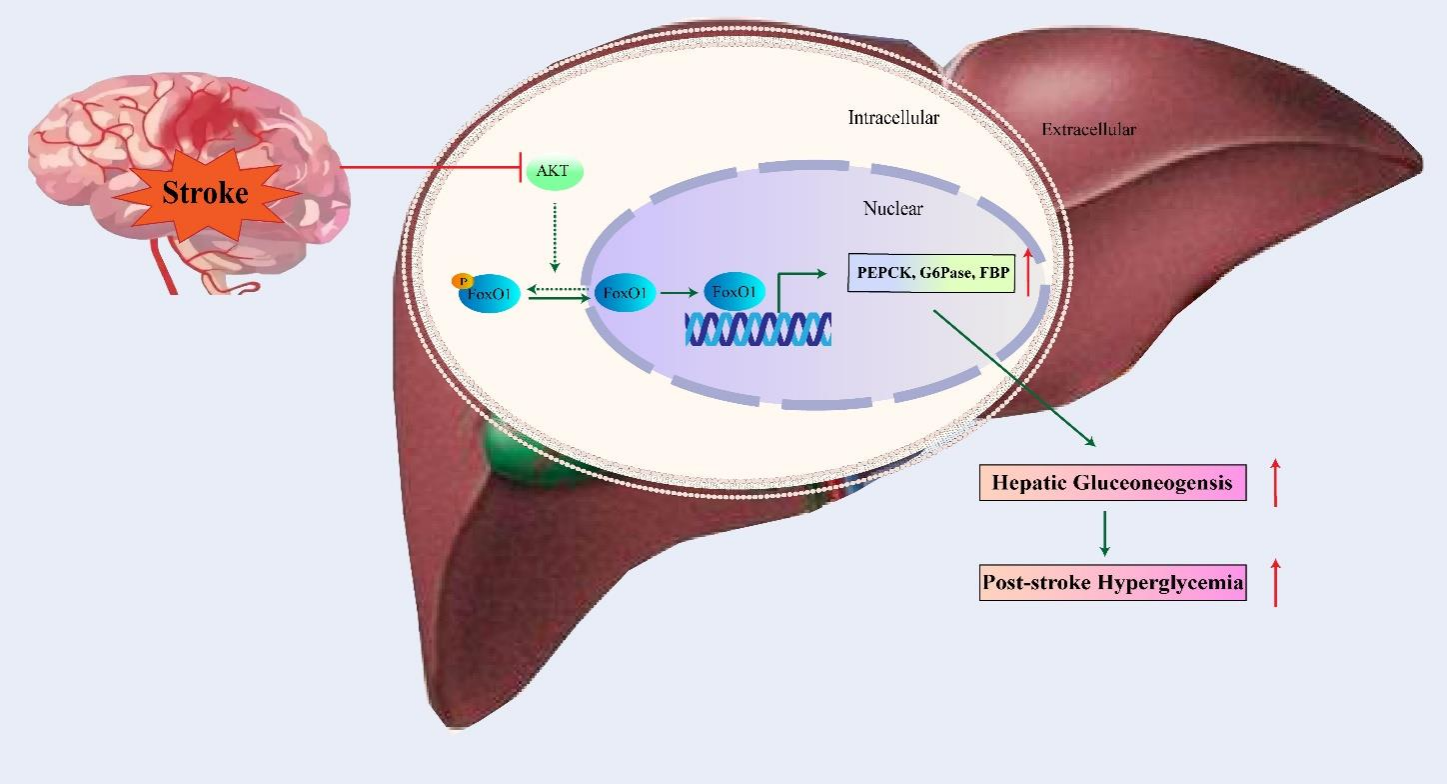
Schematic model for role of FoxO1 in hepatic gluconeogenesis. After cerebral stroke, activation of Akt pathway in liver is inhibited. Then the phosphorylation of hepatic FoxO1 is suppressed which further leads to the nuclear translocation and activation of FoxO1. Activation of FoxO1, resulting in upregulation of hepatic PEPCK, G6Pase, and FBP at the transcriptional level, facilitated hepatic gluconeogenesis and the consequences of hyperglycemia after stroke. The solid lines represent the pathway that occurs after stroke, and the dotted line represents the pathway suppressed after stroke.

FoxO1 is a well-known transcription factor for regulating hepatic gluconeogenesis[57]. After an acute ischemic stroke, approximately one-third of patients develop hyperglycemia[58]. It has been reported that within 24 hours of a stroke, rats with permanent cerebral ischemia developed increased levels of fasting blood glucose levels due to up-regulation of genes involved with hepatic gluconeogenesis through the systemic activation of glucagon, cortisol, and pro-inflammatory cytokines. FoxO1 is involved in the cortisol pathway since activation of the glucocorticoid receptor might lead to increased transcription and expression of hepatic FoxO1. Hepatic FoxO1 works with other up-regulated transcription co-activators, such as cAMP responsive element-binding protein (CREB), to promote increased gene expression of hepatic gluconeogenesis enzymes, such as phosphor-enolpyruvate carboxykinase (PCK), glucose-6-phosphatase (G6Pase), and fructose-1,6-bisphosphatase (FBP), facilitating hepatic gluconeogenesis and the consequences of hyperglycemia and its clinical consequences(Fig. 2) [59]. To summarize, the activation of FoxO1 after ischemic stroke could increase hepatic gluconeogenesis and accelerate post-stroke hyper-glycemia, resulting in a high risk of mortality.

Recently, it has been reported that increased brain gluconeogenesis occurs after cerebral ischemia and contributes to disordered glucose metabolism during ischemia and reperfusion by augmenting acidosis and oxidative damage. Moreover, increased activity of PCK showed detrimental effects in the ischemic brain [60]. Considering that FoxO1 is an important transcriptional regulator of PCK, exploring the way that FoxO1 regulates PCK and gluconeogenesis during cerebral ischemia will be a meaningful direction to explore in the future.

### FoxO1 in BBB disruption

The BBB is maintained by tight junctions (TJs) between adjacent endothelial cells inhibiting the penetration of toxic substances into the brain [61]. TJs are composed of occludin, claudin, junctional adhesion molecule (JAM), and cytoplasmic-associated proteins [62]. ZO-1 (zonula occludens-1) is an important cytoplasmic TJ-associated protein that functions by binding to occludin and recruiting it into TJs [63]. This leads to the conclusion that the loss and degradation of ZO-1 is closely linked to increased barrier permeability[64].

**Figure 3.**
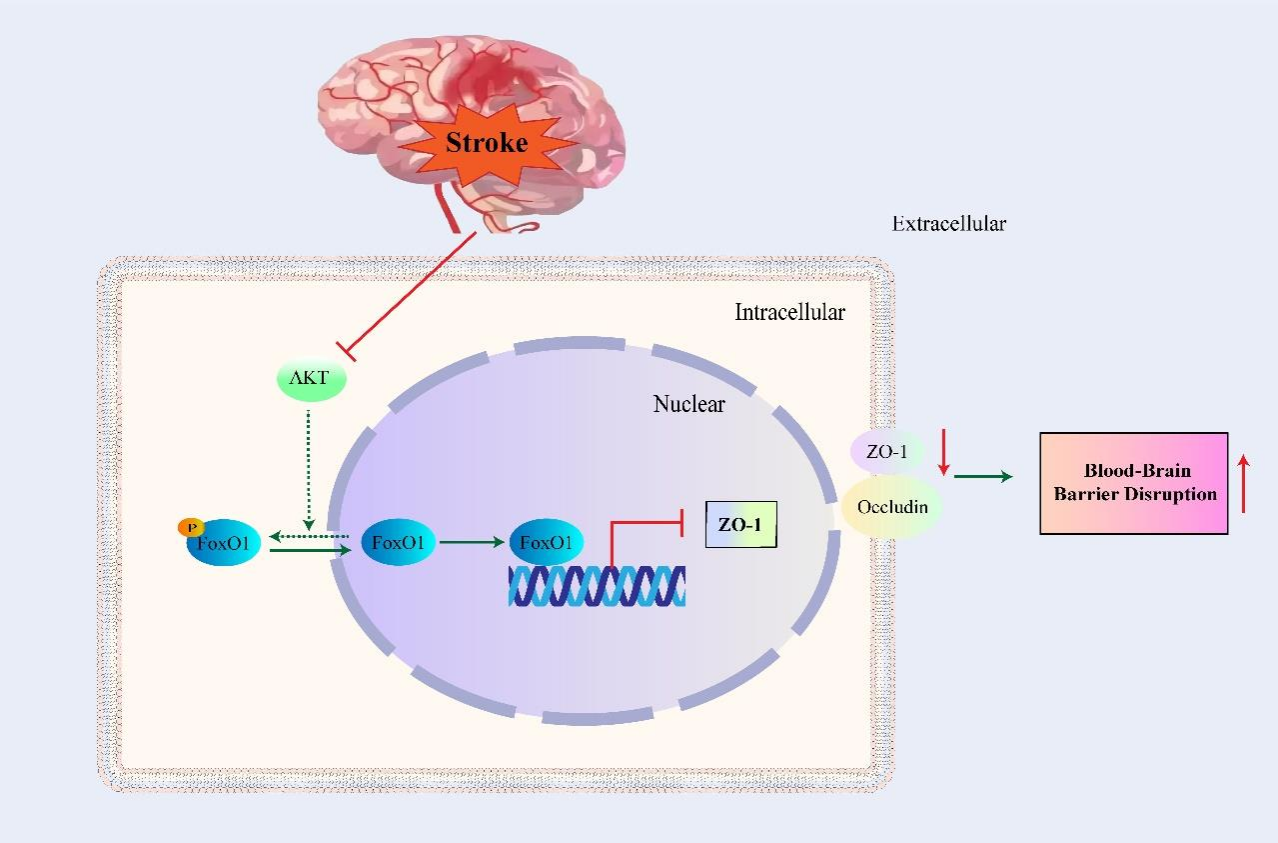
Schematic model for role of FoxO1 in BBB disruption. After stroke, activation of Akt pathway is inhibited. Then the phosphorylation of FoxO1 is suppressed which further leads to nuclear translocation and activation of FoxO1. Activation of FoxO1 results in downregulation of ZO-1 at the transcriptional level, which further leads to increased BBB disruption. The solid line represents the pathway that occurs after stroke, and the dotted line represents the pathway suppressed after stroke.

The downregulation of ZO-1 and occludin in ischemic injuries is associated with decreased expression of p-Akt and p-FoxO1 in vivo and in vitro [65]. Furthermore, FoxO1 could be activated and inhibit the expression of ZO-1 at the transcriptional level after an I/R injury, resulting in increased BBB disruption (Fig. 3) [66]. However, Zhang et al. reported that in the permanent middle cerebral artery occlusion (pMCAO) model, inhibition of FoxO1 caused the downregulation of the ratio of Bcl-2/Bax and the downregulation of ZO-1 and occludin, which resulted in endothelial cell apoptosis and BBB disruption [67].

After a subarachnoid hemorrhage (SAH), there is a noticeable disruption in the BBB. One pathway to regulate the tight junction openings and BBB permeability is the Akt/FoxO1 signaling pathway. There was an observational down-regulation in ZO-1, occludin as well as decreased p-Akt activity; and an observational increase in FoxO1 activation, resulting in BBB disruption and indicating that FoxO1 plays a key role in the destruction of BBB permeability [68].

### FoxO1 promotes inflammatory response

**Figure 4.**
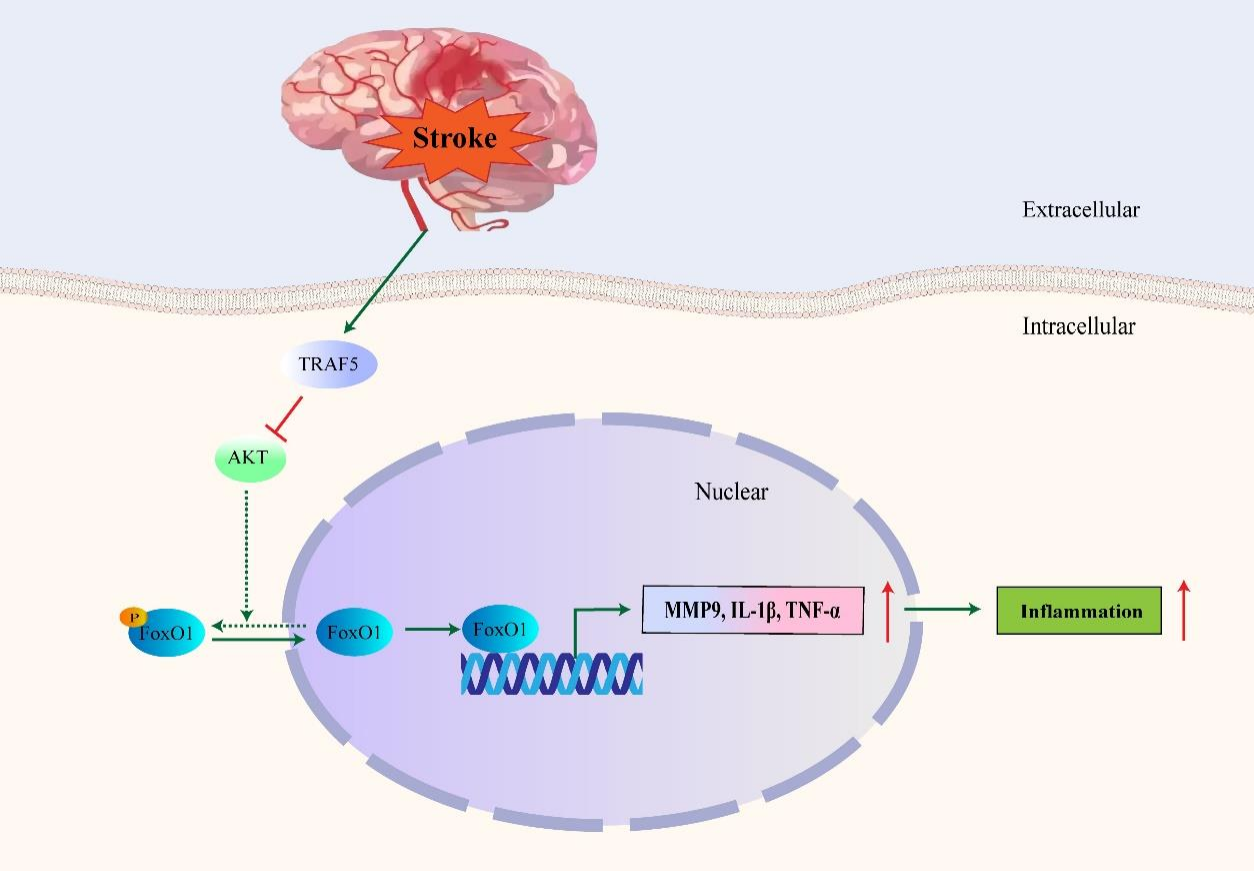
Schematic model for role of FoxO1 in inflammation. After stroke, TRAF5 is activated, leading to the suppression of Akt pathway. Then the phosphorylation of FoxO1 is inhibited which further leads to the nuclear translocation and activation of FoxO1. Activation of FoxO1, resulting in an increase of MMP9, IL-1β, TNF-α at the transcriptional level, further leads to the increase of the inflammatory response. The solid line represents the pathway that occurs after stroke, and the dotted line represents the pathway suppressed after stroke.

Numerous transcription factors are associated with inflammation. Inflammation is increased post ischemic stroke, causing an immune response that leads to increased cerebral damage. FoxO1 has central roles in regulating the expression of several genes among these transcription factors, including matrix metalloproteinase-9 (MMP-9), which is associated with brain injury and inflammation. Furthermore, high levels of thrombin are also involved in the pathological process in stroke models. Thrombin-induced MMP-9 expression is mediated via FoxO1 activation in SK-N-SH cells [69-71]. FoxO1 also participates in stimulating the expression of pro-inflammatory cytokines, including interleukin-1 beta (IL-1β), tumor necrosis factor-alpha (TNF-α), CCL20 and L-selectin [72, 73]. Knockdown or deletion of FoxO1 could attenuate inflammatory cytokine expression and inhibit the inflammatory response mediated by toll-like receptor (TLR) [74, 75]. An inhibited inflammatory response and overall protective effect has been exhibited in the ischemic brain of tumor necrosis factor receptor-associated factor 5 (TRAF5) KO mice, which was associated with the elevated phosphorylation of Akt/FoxO1. Expression of TRAF5 increased after ischemia in mice, resulting in inhibited phosphorylation of Akt as well as activation of FoxO1, which further promoted the transcriptional activation of inflammatory cytokines mediated by FoxO1. The results may suggest that decreasing activity of FoxO1, mediated by TRAF5/Akt pathway, could be associated with decreased inflammatary cytokines, and ultimately lead to decreased cell damage in cerebral I/R model (Fig. 4) [76].

Li et al. demonstrated that the expression of FoxO1 was increased following intracerebral hemorrhage (ICH) in response to stress caused by surgery and ICH. Levels of FoxO1 peaked around 12 h and gradually decreased until 72h, reaching closer to the baseline than the sham group. Expression levels of inflammatory cytokines also significantly increased at 12 h post-ICH compared with the sham operation group. In addition, the expression levels of FoxO1 were highest in the ipsilateral corpus striatum, indicating that an inflammatory response could be related to FoxO1 expression. FoxO1 could regulate many inflammatory factors, including TNF-α, IL-1β and interleukin-18 (IL-18), via the TLR4/nuclear factor kappa-B (NF-κB) signaling pathway at the start of an inflammatory reaction [10].

A FoxO1 knockdown decreased expression of downstream inflammatory cytokines in ICH injury and improved neurological function. Recently, Deng et al. reported recombinant CC chemokine ligand 17 (CCL17)-dependent CC chemokine receptor 4 (CCR4) activation alleviated neuro-inflammation through repression of FoxO1 after ICH in mice [77]. Therefore, FoxO1 might play a detrimental role through transcriptional regulation of the inflammatory response in the aftermath of an ICH-triggered cerebral injury [10].

### FoxO1 reduces oxidative injury

**Figure 5.**
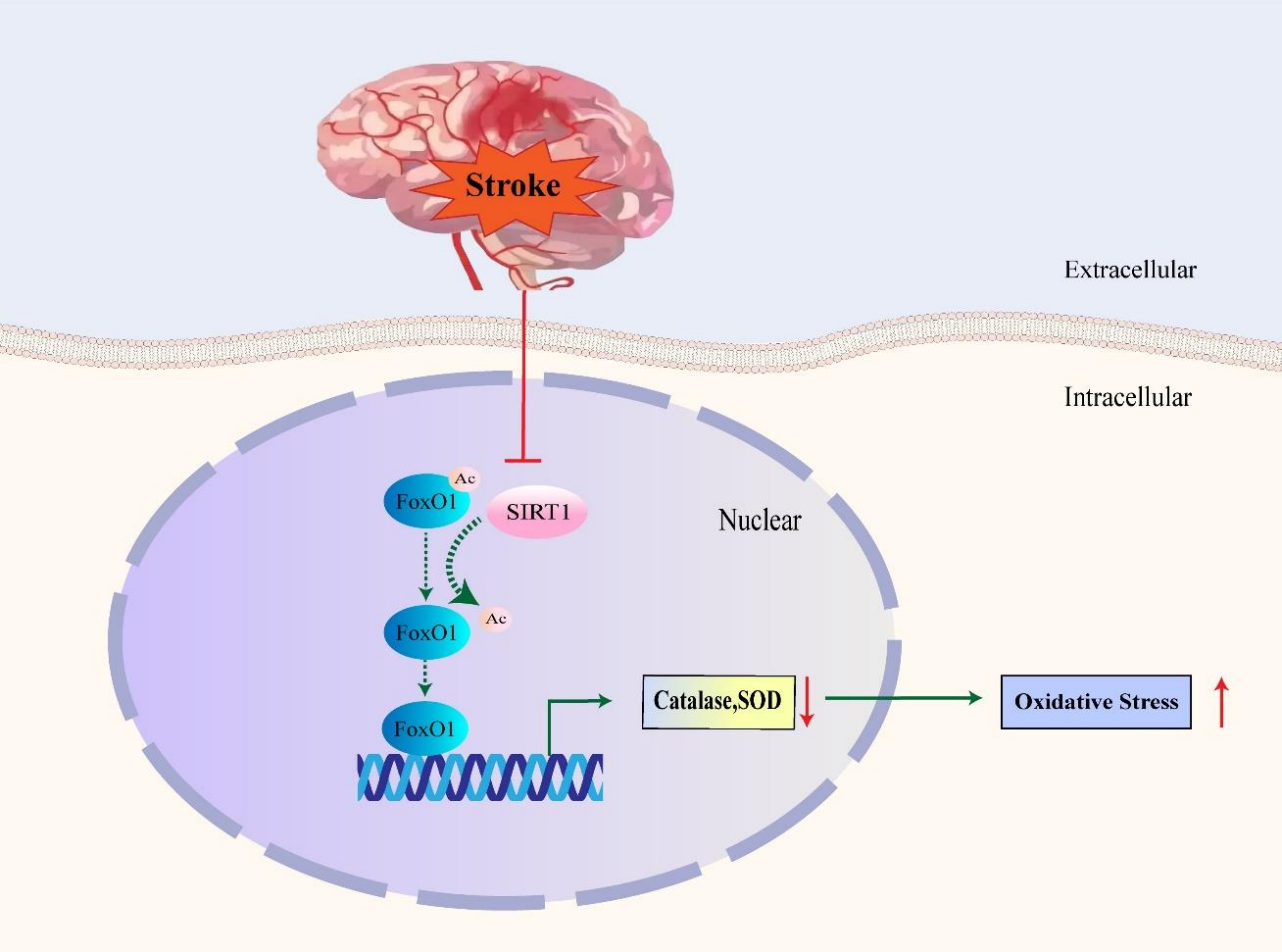
Schematic model for role of FoxO1 in oxidative injury. The SIRT1-mediated deacetylation and activation of FoxO1 can promote the synthesis of antioxidants including catalase and SOD. After stroke, the anti-oxidation pathway mediated by SIRT1 is inhibited. This may further lead to the increasing of oxidative stress. The solid line represents the pathway that occurs after stroke, and the dotted line represents the pathway suppressed after stroke.

After a stroke, the dephosphorylation of FoxO1 causes apoptosis through the Akt pathway. In contrast, deacetylation of FoxO1 has been also reported to play a neuroprotective role against neuronal apoptosis induced by oxidative stress after cerebral ischemia-reperfusion [78]. Acetylation can regulate FoxO1 transcriptional activity by changing DNA binding and promoter binding specificity [26]. It has been reported that the deacetylation regulated by SIRT1 altered the FoxO family DNA binding affinity differentially. The higher acetylation degree of FoxO3 was conducive to the expression of apoptotic proteins genes including Bim and FasL, while the deacetylated form promoted antioxidant and protective gene expression [26, 79]. Similarly, the deacetylation activation of FoxO1 through SIRT1 facilitates the synthesis of antioxidants, such as catalase and superoxide dismutase (SOD), which further induces cellular resistance against oxidative stress. The pathway is suppressed and cell will suffer from oxidative injury after an ischemic stroke (Fig. 5) [78, 80-82]. Furthermore, in carotid arteries with unstable plaques, miR-200c, a biomarker for inducing endothelial dysfunction, could negatively regulate SIRT1, de-acetylated FoxO1, and other stable biomarkers. Carotid arteries are the most prevalent region of stroke. Suppression of FoxO1 may directly lead to the reduction of these stability factors and induce an increase in ROS production [83].

FoxO1 has also been verified to be protective against ischemic strokes through the hippo signaling pathway, which controls organ size through regulation of cell proliferation, apoptosis and stem cell renewal. A protein called Yes-associated protein (YAP)1, the primary effector in the hippo signaling pathway, may directly bind to FoxO1 and upregulate its transcriptional activity, which further activates antioxidant enzymes [84]. When Yap1 is downregulated, neuronal cells suffer from increasing vulnerability to ROS species leading to eventual cell death by MCAO induction [85]. Ultimately, FoxO1 has been shown to play a neuroprotective role by protecting against ROS production, also resulting in a potential target of therapeutic effect.

## Treatment and intervention measures for FoxO1 after stroke

Since FoxO1 plays a vital role in stroke, many treatments and intervention measures targeting FoxO1 post-stroke have been tried (Table 1).

Parecoxib, which is a novel COX-2 inhibitor, functions as a neuroprotective agent and rescues neurons from cerebral ischemic reperfusion injury-induced apoptosis. The delayed administration of parecoxib may significantly inhibit the nuclear translocation of FoxO1 and its target gene-CHOP in a dose-independent manner. This would further cause inhibition of ER stress and cell apoptosis [43]. Administration of Lithium during brain ischemia has been shown to restore decreased activation of Akt 24 h after ischemia in rat brain, which is associated with increased phosphorylation of FoxO1 and protection from ischemic reperfusion injury [44].

**Table1:** Treatments and Intervention Measures Targeting FoxO1 Post-stroke.

1. Detrimental Targets of FoxO1	
Parecoxib	Significantly inhibits the nuclear translocation of FoxO1 and its target gene-CHOP in a dose-independent manner
Lithium	Restores decreased activation of Akt 24 h after ischemia, which is associated with increased phosphorylation of FoxO1
7,8-Dihydroxyflavone (7,8-DHF)	Attenuates brain tissue damage, causing Akt activation in neurons, with enhancing FoxO1 phosphorylation and decreasing the cellular apoptosis after ICH
Galangin	Inhibits the expression of MMP-9 stimulated by thrombin via blocking the FoxO1 cascade in SK-N-SH cells
KY-226	Protects the integrity of BBB by restoring the TJ protein mediated by FoxO1 inhibition, thereby protecting neurons from cerebral ischemic injury
Melatonin	Prevents the injury-induced reduction of p-Akt, p-FoxO1, and increases the interaction of p-FoxO1 with 14-3-3, which leads to reduced cell apoptosis
Bone marrow stem cells (BMSCs)	Decreases cell apoptosis and upregulates the expression of survivin, increasing phosphorylation of FoxO1
17β-estradiol	Prevents injury-induced decrease of p-Akt and p-FoxO1 during ischemia stroke
Sal (8-O-b-d-glucoside of tyrosol)	Protects against cerebral I/R injury through the inhibition of FoxO1 activation
Pre-conditioning	Prevents injury-induced decrease p-FoxO1 during ischemia stroke
2. Protective Targets of FoxO1	
OX26-PEG-Se NPs	Activates FoxO1/Catalase/SOD to inhibit oxidative injury
Calycosin-7-O-β-D-glucoside (CG)	Alleviates oxygen glucose deprivation/reoxygenation-induced damage via the inhibition production of ROS through activating of SIRT1/FoxO1 signaling pathway
Alvianolic acid B (SalB)	Upregulates the expression of SIRT1 and Bcl-2 and downregulates the expression of acetylated-FoxO1 and Bax
Piceatannol (Pic)	Plays antioxidant and neuroprotection effects via activation of SIRT1/FoxO1 pathway
Magnolol	Increases the expression of SIRT1, leading to the downregulation of Ac-FoxO1 and activation of the synthesis of antioxidants to protect against oxidative stress injury

As a member of flavonoid family compounds, 7,8-Dihydroxyflavone (7,8-DHF) has recently been identified as a specific tropomyosin-related kinase receptor B (TrkB) agonist that results in reduced brain tissue loss, neuronal damage, brain edema, and improved long-term functional outcomes after ICH. Treatment with 7,8-DHF can attenuate brain tissue damage following ICH and be proved to related with increased TrkB. This would subsequently cause Akt activation in neurons, with enhancing FoxO1 phosphorylation and decreasing the cellular apoptosis after ICH [45]. Galangin, also a flavonoid compound in flavonoids, inhibits the expression of MMP-9 stimulated by thrombin via blocking the FoxO1 cascade in SK-N-SH cells and this ultimately reduces SK-N-SH cell migration, which may further reduce brain injury [69].

KY-226 was synthesized as a potent and selective inhibitory agent with activity against protein tyrosine phosphatase 1B (PTP1B). KY-226 has been reported to protect the integrity of BBB and reduce neurological deficit by restoring the TJ protein mediated by FoxO1 inhibition, thereby protecting neurons from cerebral ischemic injury [66]. Melatonin, known as an agent that regulates circadian rhythms related to sleep and season, prevents the injury-induced reduction of p-Akt and p-FoxO1. Furthermore, the interaction of p-FoxO1 with 14-3-3 increases in the presence of melatonin, which leads to reduced cell apoptosis and infarct volume [50]. Bone marrow stem cells (BMSCs) have been shown to be protective in cerebral ischemia as well. FoxO1 has been reported to bind directly on the survivin promoter as a transcription repressor. Hypoxia-inducible factor-1 (HIF-1) α-AA is a more stable mutant form of HIF-1α, which is a crucial oxygen-sensitive regulator. Co-cultured HIF-1α-AA-modified BMSCs with neuron-like cells significantly decrease cell apoptosis and upregulate the expression of survivin, through the activation of the PI3K/Akt pathway with increasing phosphorylation of FoxO1. Afterwards, significant neurological functional recovery in rats with HIF-1α-AA-BMSCs was observed [53].

Similarly, 17β-estradiol could decrease infarct volume and prevent the injury-induced downregulation of p-Akt and p-FoxO1 during ischemic stroke [42]. Sal, an 8-O-b-d-glucoside of tyrosol, is the major bioactive component of the flower R. rosea, and it has been found to significantly protect against cerebral I/R injury by alleviating cerebral infarction, cerebral edema, and neurological deficit through the inhibition of FoxO1 activation [28]. It has been reported that pre-conditioning could protect neurons against injury from ischemia. The neuroprotective mechanism of pre-conditioning may be related to dephosphorylation of FoxO1 [86]. In conclusion, the treatment methods identified above primarily work through inhibiting FoxO1 and decreasing stroke induced cell apoptosis and brain injury, and thus, facilitating good neurological outcomes.

AS1842856 [5-amino-7-(cyclohexylamino)-1-ethyl-6-fluoro-4-oxo-1,4-dihydroquinoline-3-carboxylic acid] is a synthetic small molecule found to bind dephosphorylated FoxO1 protein in a dose dependent manner. It inhibits FoxO1 at an IC50 of 0.033mM and can potently block FoxO1 at the maximum concentration of 0.05-1mM without showing signs of cytotoxicity. The administration of AS1842856 at 0.1 M dose was also found to inhibit FoxO3a, FoxO4 and FoxO1-mediated promoter activity by 3, 20 and 70%, respectively [87, 88]. Inhibition of FoxO1 expression by AS1842856 has also been shown to attenuate renal ischemia-reperfusion injury [89], lung injury [90], and alleviates type 2 diabetes-related diastolic dysfunction[91]. As an agent that specifically targets FoxO1, the role of AS1842856 in stroke and its potential use in research and clinical application needs to be further studied.

On the contrary, activation of the SIRT1/FoxO1 pathway could protect the brain from oxidative injury and promotes good neurological outcomes during stroke. Anti-transferrin receptor monoclonal antibody (OX26)-PEGylated Se nanoparticles (OX26-PEG-Se NPs) were synthesized, and their neuroprotective effects have been shown to be associated with reducing brain edema and infarction volume through activation of the FoxO1/Catalase/SOD pathway to inhibit oxidative injury [85]. Calycosin-7-O-β-D-glucoside (CG) is a representative component of isoflavones in Radix Astragali (RA). CG alleviates oxygen glucose deprivation/reoxygenation-induced cell damage via the inhibition of ROS production through activation of SIRT1/FoxO1 signaling pathway [78]. Alvianolic acid B (SalB) is the most abundant and bioactive compound of Danshen, which is a Chinese medicinal herb. SalB plays a neuroprotective role by significantly reducing infarct volume, neurological score and brain water content through upregulating the expression of SIRT1 and Bcl-2 and downregulating the expression of acetylated-FoxO1 and Bax in experimental stroke rats [37]. Piceatannol (Pic), a natural stilbene found primarily in seeds, wines and fruits, has been shown to promote antioxidant and neuroprotective effects via activation of SIRT1/FoxO1 pathway. Pic-treated mice showed improvement in neurological and cognitive function [80]. Magnolol, found in the bark of M. grandiflora, can also increase the expression of SIRT1, leading to the downregulation of Ac-FoxO1 and activation of the synthesis of antioxidants to protect against oxidative stress injury. It is also shown to result in a significant decrease in brain water content and brain infarct volume as well as neurological score [81]. These results may further indicate the neuroprotective features of de-acetylated FoxO1 through the activation of antioxidases after stroke. Ideally, there would be downregulation of FoxO1, with upregulation of the FoxO1/SIRT1 pathway for maximum therapeutic intervention.

## Perspective and Prospective

FoxO1 has been shown to be involved in the regulation of apoptosis, anti-oxidative stress, BBB disruption, hepatic gluconeogenesis and inflammation during the pathophysiological processes of stroke. The role of FoxO1 on its target factors implies that if we could clearly clarify its regulatory activity, the FoxO1 might be a new optimized pathway of targeted stroke therapy. It is worth noting that FoxO1 not only has the ability to resist oxidative stress and help cell survival, but also can cause cell death due to its pro-apoptotic ability. It is not clear whether all the targets of FoxO1 are modulated during stroke. Moreover, the specific regulation methods of FoxO1 are still unknown in the stroke period. Different interventions targeting FoxO1 after stroke have achieved different effects. Whether these different effects are caused by the difference in treatment level or treatment time is still unclear. To solve these problems may be an important breakthrough for the molecular therapy of stroke.

In addition, FoxO1 acts as a node for multiple signaling axes. The balance betwwen the intracellular transcriptional activity of FoxO1 and its regulators is critical to stroke progression, even though its mechanism has not yet been fully established. Moreover, the phosphorylation/acetylation of FoxO1 and their interactions need to be further explored. In conclusion, the roles of FoxO1 within the full spectrum of stroke, as well as the subtle regulation and application mechanism of FoxO1, are waiting to be studied and defined. This will ultimately identify effective drugs and accurate usage methods, allowing potent treatments for debilitating conditions such as stroke or CAH.
